# Aspirin (Acetylsalicylic Acid) Exerts Antineoplastic Effects on Bile Duct Carcinoma Cells Through Modulation of COX-2/EGFR, AMPK, and IGF-1R Signaling Pathways

**DOI:** 10.5152/tjg.2026.25775

**Published:** 2026-05-04

**Authors:** Yoonchan Lee, Jin Lee, Eun Mi Hong, Kyong Joo Lee, Se Woo Park, Dong Hee Koh

**Affiliations:** 1 Division of Gastroenterology, Department of Internal Medicine, University of Ulsan College of Medicine, Asan Medical Center, Seoul, Republic of Korea; 2 Division of Gastroenterology, Department of Internal Medicine, Hallym University Dongtan Sacred Heart Hospital, Hallym University College of Medicine, Hwasung, Republic of Korea

**Keywords:** AMP-activated protein kinase, aspirin, bile duct neoplasm, cyclooxygenase-2, epidermal growth factor receptor, epithelial–mesenchymal transition

## Abstract

**Background/Aims:**

: Bile duct carcinoma (BDC) is a highly aggressive malignancy. While epidemiological evidence suggests that acetylsalicylic acid (ASA [aspirin]) reduces BDC risk, the underlying molecular mechanisms have not been fully elucidated. This investigation explored the antineoplastic mechanisms of ASA in BDC cells.

**Materials and Methods:**

: The human BDC cell line SNU-245 was used in all experiments. Cell viability was determined using 3-(4,5-dimethylthiazol-2-yl)-2,5-diphenyltetrazolium bromide assays, whereas apoptosis and caspase-3 levels were evaluated using enzyme-linked immunosorbent assay. Protein expression was analyzed using Western blotting and immunofluorescence. Functional pathway interactions were investigated using siRNA-mediated gene silencing.

**Results:**

: ASA reduced cell viability and increased apoptosis markers, accompanied by increased Bax and p53 expression and decreased Bcl-2 levels. ASA treatment reduced cyclooxygenase-2 (COX-2) expression and decreased epidermal growth factor receptor (EGFR) levels. COX-2 knockdown markedly attenuated deoxycholic acid (DCA)-induced EGFR phosphorylation, whereas EGFR silencing partially reduced DCA-induced COX-2 expression. These results suggest reciprocal signaling interactions, with COX-2 exerting a relatively stronger upstream influence. ASA increased phosphorylation of AMP-activated protein kinase at threonine 172 (AMPKᵀʰʳ¹⁷²) and reduced insulin-like growth factor-1 receptor (IGF-1R)/insulin receptor substrate-1 (IRS-1) signaling and decreased mammalian target of rapamycin (mTOR) phosphorylation. ASA also attenuated epidermal growth factor (EGF)-induced changes in epithelial–mesenchymal transition–related markers, including preservation of E-cadherin and reduction of N-cadherin expression.

**Conclusion:**

: In this in vitro model, ASA exposure was associated with coordinated modulation of multiple cell-survival–related signaling pathways in BDC cells, including COX-2/EGFR signaling, AMPK activation, and IGF-1R–mediated mTOR regulation. These findings provide mechanistic insight into the potential antineoplastic effects of ASA and support further translational studies in BDC.

**Main Points**

- Acetylsalicylic acid (ASA) induces apoptosis and reduces proliferation in bile duct carcinoma cells.
- ASA suppresses deoxycholic acid–associated activation of cyclooxygenase-2 and epidermal growth factor receptor (EGFR) signaling cascades.
- ASA enhances AMPKᵀʰʳ¹⁷² phosphorylation and inhibits IGF-1R/IRS-1 signaling, thereby promoting TSC-2 activation and suppressing mammalian target of rapamycin.
- ASA reverses epithelial–mesenchymal transition under EGF stimulation by restoring E-cadherin expression and reducing N-cadherin expression.

## Introduction

Bile duct carcinoma(BDC) is characterized by delayed symptom onset and limited opportunities for early detection.^1^ Use of acetylsalicylic acid (ASA) for over 6 years reduced colorectal cancer mortality,^2^ and a follow-up study demonstrated that long-term ASA use improves survival, particularly in patients with tumors exhibiting high cyclooxygenase-2 (COX-2) expression.^3^ A meta-analysis reported that prolonged ASA use significantly reduces overall and gastrointestinal cancer mortality.^4^

The impact of ASA on BDC risk remains controversial.^5^ Nevertheless, a case–control study demonstrated that ASA reduces the risk of developing all types of BDC by 2.7- to 3.6-fold.^6^ A meta-analysis further revealed that ASA users had a decreased risk of BDC, with a more pronounced reduction in intrahepatic than extrahepatic cholangiocarcinoma.^7^ Further investigations have reported reduced mortality from biliary malignancies, including gallbladder cancer, intrahepatic cholangiocarcinoma, ampulla of Vater cancer, and hilar cancer,^8^ supporting the potential anticancer effects of ASA. Although the precise mechanisms by which ASA exerts its antineoplastic effects remain elusive, COX-2 inhibition is considered a major pathway. COX-2 expression is increased in chronically inflamed tissues, promoting tumorigenesis via several pathways, including epidermal growth factor receptor (EGFR) signaling.^9^ Additionally, bile acids activate EGFR, inducing p42/44 mitogen-activated protein kinase (MAPK) and p38 MAPK signaling, thereby further enhancing COX-2 expression.^10^ However, a meta-analysis reported that other nonsteroidal anti-inflammatory drugs targeting COX-2 did not demonstrate significant antineoplastic effects,^11^ suggesting that ASA may act through other mechanisms.

Recent studies have demonstrated that metformin inhibits BDC proliferation by activating AMP-activated protein kinase, inhibiting mammalian target of rapamycin (mTOR) signaling, and suppressing insulin-like growth factor-1 receptor (IGF-1R)/Akt pathways.^12^ Previous investigations have shown that ASA inhibits serine phosphorylation of insulin receptor substrate-1 (IRS-1) and suppresses mTOR signaling through AMPK activation.^13^ However, the underlying mechanisms have not been further investigated in BDC cells.

Therefore, this study evaluated the in vitro effects of ASA on BDC cells, with a focus on signaling pathways involving COX-2, EGFR, and IGF-1R, particularly under stimulation by toxic bile acid and growth factors. Furthermore, we aimed to investigate the effects of ASA on epithelial–mesenchymal transition (EMT), a key process involved in tumor aggressiveness.

## Materials and Methods

### Materials

Roswell Park Memorial Institute (RPMI)-1640 medium containing 10-mM glucose, phosphate-buffered saline (PBS), penicillin/streptomycin, trypsin–ethylenediaminetetraacetic acid (EDTA), and fetal bovine serum (FBS) were obtained from Gibco (Grand Island, NY, USA). ASA, deoxycholic acid (DCA), 3-(4,5-dimethylthiazol-2-yl)-2,5-diphenyltetrazolium bromide (MTT), and dimethyl sulfoxide (DMSO) were purchased from Sigma-Aldrich (St. Louis, MO, USA). Primary antibodies against Bcl-2-like protein 4 (Bax), B-cell lymphoma 2 (Bcl-2), TSC-2, phospho-mTOR (Ser2448), mTOR, phospho-AMPK, AMPK, E-cadherin, N-cadherin, and β-actin were obtained from Cell Signaling Technology (Danvers, MA, USA). Antibodies against phospho-IRS-1, IRS-1, pro-IGF-1R, and horseradish peroxidase (HRP)-conjugated goat anti-rabbit IgG were purchased from Santa Cruz Biotechnology (Santa Cruz, CA, USA). Protein bands were visualized using a chemiluminescent HRP substrate (Takara, Kusatsu, Shiga, Japan).

### Cell Culture and Ethics Statement

The human BDC cell line SNU-245 was obtained from the Korean Cell Line Bank (KCLB) and authenticated by short tandem repeat analysis.^14^ Cells were cultured in RPMI-1640 medium supplemented with 10% FBS, 100 IU/mL penicillin, 100 μg/mL streptomycin, 2 mM glutamine, and 1.5 g/L sodium bicarbonate. The medium was replaced twice weekly, and the cells were maintained at 37°C in 5% CO₂. Upon reaching confluence, cells were passaged using trypsin–EDTA solution (2.5 g/L trypsin and 1 g/L EDTA). Ethical approval was waived by the Ethics Committee of Hallym University Dontan Sacred Heart Hospital (HDT NON2021-003; August 4, 2021), as this study used a commercially available cell line obtained from KCLB. Therefore, informed consent was not required.

### 3-(4,5-Dimethylthiazol-2-yl)-2,5-Diphenyltetrazolium Bromide Assay

Cell proliferation was assessed using an MTT assay. Cells were seeded in 96-well plates (5 × 10⁴ cells/mL) for 24 hours. Cells were then exposed to graded concentrations of ASA for 24-72 hours. A working solution of MTT (0.5 mg/mL) was added and incubated for 4 hours at 37°C. Formazan crystal was dissolved in 100 μL of DMSO, and the absorbance was measured at 570 nm using a microplate reader (ELS800, BioTek Instruments, Winooski, VT, USA).

### Caspase-3 Activity Assay

Caspase-3 activity was evaluated using a commercial kit (BioVision, Mountain View, CA, USA) according to the manufacturer’s guidelines. Cells were seeded (2 × 10⁶ cells/mL) and treated with ASA for 24-72 hours. Cells were then rinsed, lysed, and maintained on ice for 10 minutes. The lysate was then clarified by high-speed centrifugation (15 000 × *g* at 4°C), and the supernatant was collected for protein quantification using the Bradford assay (Sigma-Aldrich). A reaction mixture composed of 90 μg of total protein in assay buffer and 5 μL of the chromogenic substrate (4 mM aspartic acid-glutamic acid-valine-aspartic acid-p-nitroaniline (DEVD-pNA)) was transferred into 96-well plates and incubated for 2 hours at 37°C. The absorbance was measured at 405 nm.

### Cell Apoptosis Assay

Apoptotic cell death was assessed using a Cell Death Detection enzyme-linked immunosorbent assay (ELISA) Kit (Roche, Mannheim, Germany), which detects DNA fragments bound to histones. Cells were seeded at 2 × 10⁴ cells/mL, allowed to equilibrate for 24 hours, and exposed to ASA at the indicated concentrations. The apoptosis ELISA assay was performed at 48 and 72 hours, based on preliminary MTT and caspase-3 activity results, which demonstrated moderate apoptotic changes at 24 hours and more pronounced effects at later time points. After treatment, cells were lysed with 100 μL of lysis buffer for 30 minutes on ice, and lysates were cleared by centrifugation (15 000 × *g*, 10 minutes, 4°C). The supernatant was transferred to streptavidin-coated microplate wells and incubated with anti-DNA–peroxidase(POD) and biotinylated anti-histone antibodies for 2 hours at room temperature. After washing, 100 μL of ABTS substrate solution was added, and color development was allowed to proceed for 20 minutes. The absorbance was recorded at 405 nm.

### Transfection of siRNA

BDC cells were seeded in 6-well culture plates at 5 × 10⁴ cells/mL and grown to 40%–50% confluence. Cells were transfected with synthetic siRNA oligonucleotides targeting COX-2, EGFR, IGF-1R, and AMPK, along with a negative control siRNA (Invitrogen, Carlsbad, CA, USA). Transfection was performed using 20 pmol of siRNA in 500 µL of serum-free OPTI-MEM (Gibco) complexed with 3 µL Lipofectamine 2000 (Invitrogen). Transfection was carried out for 48 hours in 1.5 mL of antibiotic-free growth medium. After transfection, cells were washed and incubated for an additional 24 hours in serum-free medium containing 1% BSA (Sigma-Aldrich). Silencing efficacy was confirmed by immunoblotting.

### Western Blot Assay

Western blot analysis was performed as previously described.^12^ Briefly, cellular cultures at 80% confluence were treated with varying concentrations of ASA, DCA, EGF, or IGF-1 for 24, 48, or 72 hours. The cells were then harvested, rinsed, and lysed using radioimmunoprecipitation assay buffer (RIPA) buffer (Sigma-Aldrich) with subsequent centrifugation (15 000 × *g* , 20 minutes). Protein concentrations were determined using the Bradford assay. After membrane transfer, nonspecific binding sites were blocked at room temperature, and the blots were subsequently incubated overnight at 4°C with primary antibodies against Bcl-2, Bax, p53, phospho-AMPKᵀʰʳ¹⁷², total AMPK, COX-2, phospho-EGFR, EGFR, pro-IGF-1R, phospho-IRS-1, total IRS-1, TSC-2, phospho-mTOR, mTOR, N-cadherin, E-cadherin, and β-actin. Antibodies were used at 1 : 1000 dilution, except pro-IGF-1R and IRS-1 (1 : 500). Following washes with tris-buffered saline with tween 20 (TBS-T), the membranes were incubated with an HRP-conjugated goat anti-rabbit secondary antibody for 30 minutes at room temperature. Protein bands were then detected using the Hyper HRP substrate for Western blotting, and densitometric analysis was performed using ImageJ (NIH, version 1.43).

### Immunofluorescence Staining Assay

For protein localization, cells were grown on glass coverslips and treated with ASA or EGF for 24 hours. Cellular architecture was preserved by fixation with 4% paraformaldehyde (10 minutes) followed by blocking with 1% albumin and goat serum for 1 hour at room temperature. Cells were then incubated with the E-cadherin antibody in PBS with 1% BSA for 1 hour. Secondary detection was performed using FITC-conjugated immunoglobulin in PBS containing 1% FBS for 1 hour followed by 3 washes with PBS. Nuclei were stained with 4′,6-diamidino-2-phenylindole (DAPI, 0.5 μg/mL) for 1 minute, and images were acquired using super-resolution confocal laser microscopy (Carl Zeiss, Oberkochen, Germany).

### Statistical Analysis

All experiments were performed in triplicate, and the results are expressed as mean ± SD from at least 3 independent experiments. Normality was assessed using skewness and kurtosis. Multiple group comparisons were performed using 1-way analysis of variance with subsequent Tukey post-hoc testing, whereas binary comparisons were performed using the Student’s *t*-test. Computational analyses were performed using IBM-Statistical Package for the Social Sciences (SPSS) , version 27 (IBM SPSS Corp.; Armonk, NY, USA), and *P* < .05 was considered statistically significant.

## Results

### Acetylsalicylic Acid Inhibits Bile Duct Carcinoma Cell Proliferation and Promotes Apoptotic Cell Death

**Figure 1:**
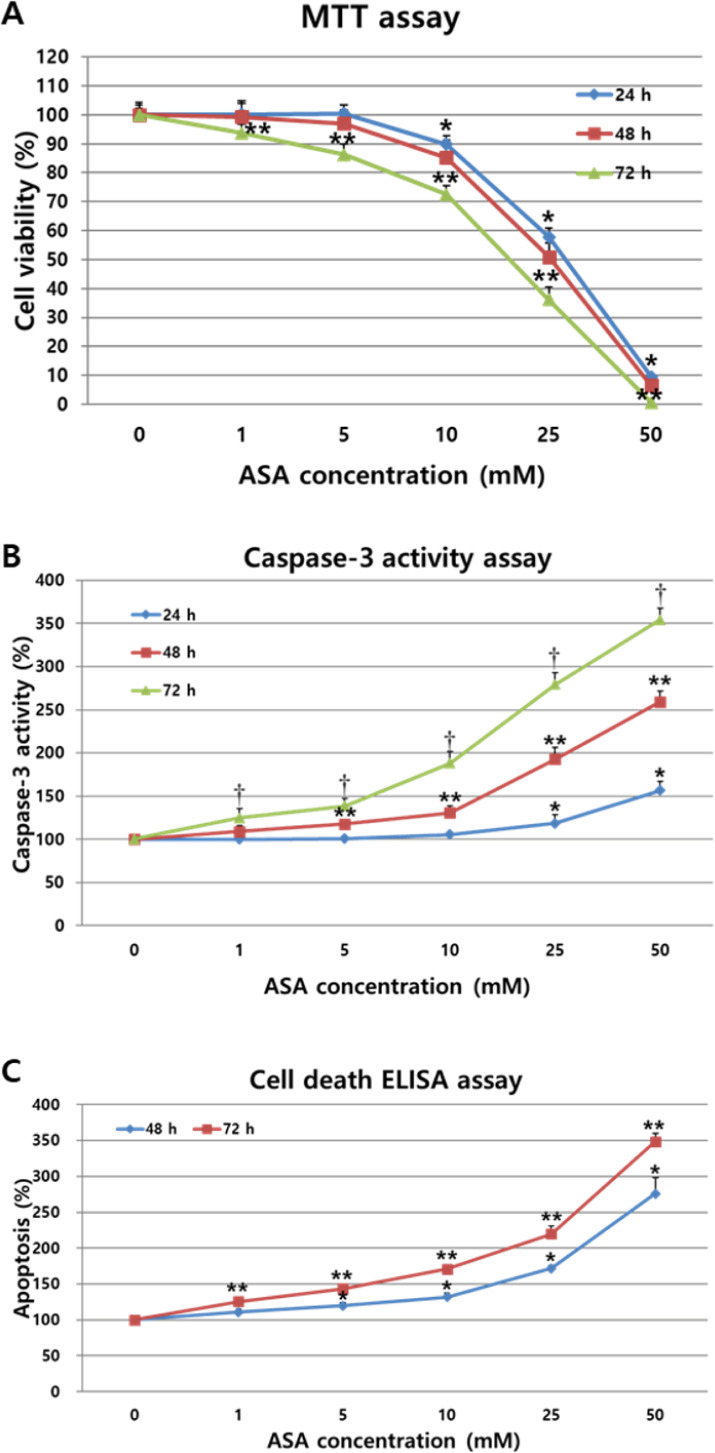
ASA inhibits proliferation and induces apoptosis in BDC cells in a dose- and time-dependent manner. (A) Cell viability of SNU-245 BDC cells following treatment with increasing concentrations of ASA (0, 2.5, 5, 10, 25, and 50 mM) was assessed using the MTT assay after 24, 48, and 72 hours of incubation. *P < .001 and **P < .001 vs. untreated control cells and cells treated with the next lower concentration of ASA for 24 and 48 hours. (B) Caspase-3 activity in BDC cells treated with ASA (0, 2.5, 5, 10, 25, and 50 mM) for 24, 48, and 72 hours was measured using a colorimetric assay kit, as described in the section “Materials and Methods.” *P < .01 and **P < .01 vs. untreated control cells and cells treated with the next lower concentration of ASA for 24 hours; ^†^*P* < .01 vs. untreated control cells and cells treated with the next lower concentration of ASA for 24 and 48 hours. (C) Apoptosis in BDC cells treated with ASA (0, 2.5, 5, 10, 25, and 50 mM) for 48 and 72 hours was quantified using the Cell Death Detection ELISA assay. *P < .01 and **P < .01 vs. untreated control cells and cells treated with lower concentrations of ASA for 48 hours. ASA, acetylsalicylic acid; BDC, bile duct carcinoma; MTT, 3-(4,5-dimethylthiazol-2-yl)-2,5-diphenyltetrazolium bromide.

To determine whether ASA affects BDC cell viability, an MTT assay was performed following 24-72 hours of treatment. ASA was associated with reduced cell viability in a concentration- and time-dependent manner (Figure 1A). Caspase-3 activity, a central mediator of apoptotic cell death, was quantified using a Caspase-3 ELISA Assay Kit, as described in the “Materials and Methods” section. Consistent with the MTT findings, caspase-3 activity was increased in association with longer exposure and higher ASA concentrations (Figure 1B). On the basis of these time-dependent patterns, DNA fragmentation was further assessed using an apoptosis ELISA at 48 and 72 hours, when the apoptotic signal was more pronounced (Figure 1C). Collectively, these data indicated that ASA exposure is associated with reduced proliferative capacity and increased apoptotic markers in BDC cells in a dose- and time-dependent manner.

### Acetylsalicylic Acid Enhances Bax and p53 Expression and Inhibits Bcl-2 Expression in Bile Duct Carcinoma Cells

To determine whether ASA modulates major apoptosis-related proteins, the expression levels of Bax, Bcl-2, and p53 were examined by Western blotting following 48 hours (Supplementary Figure 1A) and 72 hours (Supplementary Figure 1B) of treatment. ASA increased Bax and p53 expression while concurrently reducing Bcl-2 levels in a dose-dependent manner (Supplementary Figure 1). These observations were consistent with a shift toward proapoptotic signaling in ASA-treated BDC cells.

### Acetylsalicylic Acid Reduces Cyclooxygenase-2 and Epidermal Growth Factor Receptor Expression in Bile Duct Carcinoma Cells

**Figure 2:**
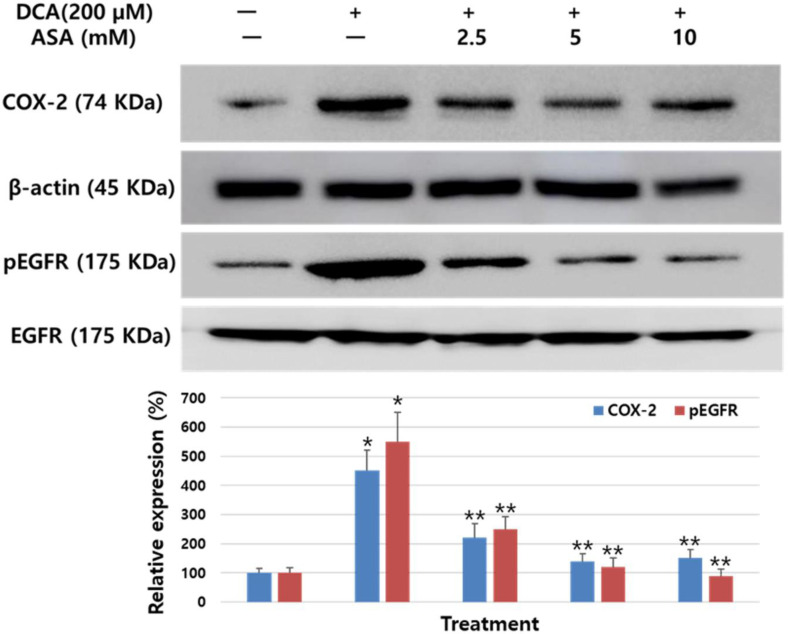
ASA counteracts DCA-induced upregulation of COX-2 and pEGFR expression in BDC cells. BDC cells were pretreated with increasing concentrations of ASA (0, 2.5, 5, and 10 mM) for 18 hours, followed by co-treatment with DCA (200 μM) for an additional 6 hours to mimic toxic bile acid exposure in BDC. Western blotting was then performed to assess COX-2 and EGFR protein expression. *P < .001 vs. untreated control (compared to respective proteins); **P < .001 vs. DCA-treated cells without ASA (compared to respective proteins). ASA, acetylsalicylic acid; BDC, bile duct carcinoma; COX-2, cyclooxygenase-2; DCA, deoxycholic acid; EGFR, epidermal growth factor receptor; pEGFR, phosphorylated EGFR.

Changes in COX-2 and EGFR expression, which are strongly associated with BDC tumor growth, were evaluated by Western blot analysis following 18 hours of ASA pretreatment and subsequent treatment with DCA (200 μM), a toxic bile acid known to activate COX-2 and EGFR, for an additional 6 hours.^15^ Notably, ASA significantly suppressed DCA-induced COX-2 and EGFR expression in a concentration-dependent manner compared to DCA-treated cells (Figure 2). These observations suggest that ASA exposure influences COX-2/EGFR-related signaling, thereby limiting the proliferative capacity of BDC cells.

### Epidermal Growth Factor Receptor Activation Appears To Be Necessary for Cyclooxygenase-2 Expression, Whereas Enhanced Cyclooxygenase-2 Expression Plays a Critical role in Epidermal Growth Factor Receptor Phosphorylation in Bile Duct Carcinoma Cells

**Figure 3:**
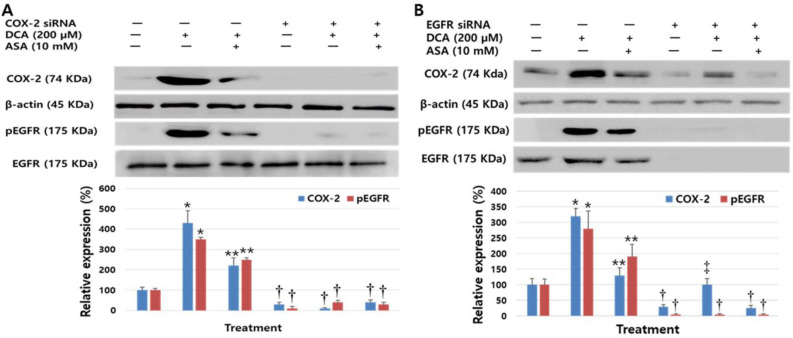
EGFR phosphorylation appears to contribute to full COX-2 activation, whereas COX-2 activity appears to contribute to EGFR activation in BDC cells. Cells were pretreated with the indicated concentration of ASA for 18 hours and then co-treated with DCA (200 μM) for 6 hours. As described in the “Materials and Methods” section, the cells were then transfected with siRNA against COX-2 (A) and EGFR (B) and incubated for 4 hours. Expression levels were analyzed using Western blotting. *P < .001 vs. untreated control; **P < .001 vs. only DCA-treated cells; ^†^*P* < .001 vs. only DCA-treated cells and ASA + DCA–treated cells without siRNA silencing; ^‡^*P* < .001 vs. only DCA-treated cells (*P* > .05 vs. ASA/DCA-treated cells without siRNA silencing) for COX-2 expression. BDC, bile duct carcinoma; COX-2, cyclooxygenase-2; DCA, deoxycholic acid; EGFR, epidermal growth factor receptor.

Upon pretreatment with or without 10 mM ASA for 18 hours, followed by an additional 6-hour exposure with 200 μM DCA, the expression levels of COX-2 and phosphorylated EGFR (pEGFR) were evaluated following siRNA transfection targeting COX-2 or EGFR to investigate the interaction between these 2 proteins. Changes in pEGFR expression after siRNA transfection for COX-2 showed that COX-2 silencing almost completely inhibited DCA-induced pEGFR expression (Figure 3A). However, EGFR silencing only partially inhibited DCA-induced COX-2 expression (Figure 3B). These results suggest that EGFR activation contributes to COX-2 expression, whereas COX-2 exerts a stronger influence on DCA-induced EGFR phosphorylation under the present conditions, indicating that COX-2 may represent a key modulatory node in BDC cells.

### Acetylsalicylic Acid Interferes with Insulin-Like Growth Factor-1 Receptor/Insulin Receptor Substrate-1-Mediated Inhibition of TSC-2, Thereby Suppressing Mammalian Target of Rapamycin Phosphorylation

We assessed the impact of ASA on the IGF-1R/IRS-1 pathway, which negatively regulates TSC-2 and promotes mTOR activation.^13^ ASA was associated with reduced IGF-1R (α/β) expression and decreased IRS-1 phosphorylation, accompanied by increased TSC-2 expression and reduced mTOR phosphorylation in BDC cells (Supplementary Figure 2A). IGF-1R knockdown recapitulated these effects, increasing TSC-2 levels and reducing phosphorylated mTOR (pmTOR) even in the absence of ASA (Supplementary Figure 2B). These findings supported the role of IGF-1R as an upstream regulator of the TSC-2/mTOR axis in BDC cells and suggested that the modulatory effect of ASA on this pathway may be mediated, at least in part, through IGF-1R.

### AMPK^Thr172^ Phosphorylation Is Associated with Changes in TSC-2 and Mammalian Target of Rapamycin Signaling in Acetylsalicylic Acid-Treated Bile Duct Carcinoma Cells

**Figure 4:**
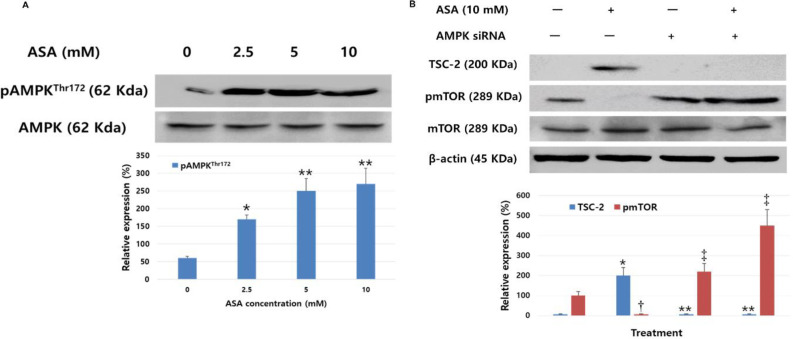
AMPK^Thr172^ plays a key role in modulating TSC-2 and pmTOR expression in BDC cells. (A) BDC cells were treated with ASA (0, 2.5, 5, and 10 mM) for 72 hours. Phosphorylation of AMPK at threonine 172 was assessed by Western blotting. *P < .001 vs. untreated control cells, **P < .01 vs. untreated control cells and cells treated with 2.5 mM concentration of ASA. (B) BDC cells were transfected with AMPK siRNA for 4 hours, followed by treatment with ASA (10 mM) for 72 hours. TSC-2 and mTOR protein expression were assessed by Western blotting. *P < .001 vs. untreated control cells and cells treated with lower concentrations of ASA for TSC-2; **P < .001 vs. only ASA-treated cells for TSC-2; ^†^*P* < .001 vs. untreated control for mTOR; ^‡^*P* < .001 vs. untreated control and only ASA-treated cells for mTOR expression. ASA, acetylsalicylic acid; BDC, bile duct carcinoma.

The current study examined the effect of ASA on AMPK phosphorylation, as activation of AMPK^Thr172^ has been reported to stimulate TSC-2 and suppress mTOR signaling.^12^ ASA increased the phosphorylation of AMPK^Thr172^ in a concentration-dependent manner in BDC cells (Figure 4A). To determine whether AMPK Thr172 plays a functional role in this process, AMPKα expression was silenced using siRNA transfection. AMPKα knockdown suppressed ASA-induced TSC-2 activation and reactivated pmTOR that had been inhibited by ASA in BDC cells (Figure 4B), suggesting that AMPK^Thr172^ phosphorylation contributes to the regulation of TSC-2 and mTOR signaling in these cells.

### Acetylsalicylic Acid Inhibits Epithelial–Mesenchymal Transition in Bile Duct Carcinoma Cells

To determine whether ASA affects EMT, immunofluorescence analysis was performed to examine E-cadherin localization after ASA and EGF co-treatment. ASA (10 mM) prevented the EGF-induced loss of membrane E-cadherin after 72 hours of exposure (Supplementary Figure 3A). Consistent with these observations, Western blotting demonstrated that EGF reduced E-cadherin and increased N-cadherin expression, whereas ASA restored E-cadherin and suppressed N-cadherin under the same conditions (Supplementary Figure 3B). Collectively, these results suggested that ASA counteracts EGF-driven EMT and helps maintain epithelial characteristics in BDC cells.

## Discussion

ASA, classified as a nonsteroidal anti-inflammatory drug, induces suppression of COX enzymes by acetylating serine residues (Ser529 in COX-1 and Ser516 in COX-2).^16^ A previous study demonstrated that COX-2 expression is stronger and more homogeneous in BDC cells than in non-neoplastic epithelial cells.^15^ Hydrophobic bile acids such as DCA stimulate both EGFR and COX-2, initiating cascades involving MAPK and JNK, as well as phosphoinositide 3-kinase/protein kinase B (PI3K/Akt) signaling pathways that drive proliferation, survival, invasion, and angiogenesis.^13^ Because these pathways contribute to BDC pathogenesis, regulating COX-2 and EGFR activity represents a promising therapeutic target.^17^ The current study found that ASA treatment reduced DCA-induced COX-2 and EGFR expression in BDC cells in a concentration-dependent manner, restoring their expression to levels prior to DCA stimulation. Furthermore, the authors found that ASA induced apoptosis and inhibited the proliferation in BDC cells by enhancing Bax and p53 expression while downregulating Bcl-2. These findings suggested that ASA induces apoptosis in BDC cells by both suppressing EGFR and COX-2 signaling and directly modulating apoptotic regulators, thereby providing multiple complementary mechanisms for its antineoplastic effects. However, further studies using gene-silenced cells are warranted to confirm whether ASA-induced apoptosis is dependent on these pathways.

A previous study reported that ASA induced cell cycle arrest but did not trigger apoptosis in BDC cells.^18^ In the present study, ASA exposure was associated with apoptotic changes in BDC cells. These differences may reflect variations in experimental conditions, including drug concentration, exposure time, or cellular models. Beyond these differences in cellular responses, the current study expands upon prior reports by examining the simultaneous modulation of multiple oncogenic signaling pathways, including COX-2/EGFR crosstalk, IGF-1R/IRS-1–related TSC-2 regulation, AMPK-associated mTOR signaling, and changes in EMT-related markers. In addition to these signaling pathways, several studies have reported that ASA modulates p53 pathway in various cancer types. For example, ASA has been shown to enhance p53 expression and activity in colorectal cancer cells, thereby contributing to growth inhibition and apoptosis.^13^ Moreover, ASA-mediated regulation of p53 signaling has been described in hepatocellular carcinoma and gastric cancer models, where increased p53 levels were associated with antiproliferative effects.^19^^,^^20^ Consistent with these findings, the current study demonstrated that a dose-dependent increase in p53 expression following ASA treatment in BDC cells, supporting a potential role for p53 modulation in the observed antitumor effects.

Earlier studies investigating the interaction between COX-2 and EGFR in other cancer types have shown that COX-2-produced prostanoids can activate EGFR kinase in colon cancer cells, whereas EGFR activation has been reported to upregulate COX-2 expression.^21^ The siRNA-mediated silencing experiments in the current study provided important insights into this relationship in BDC cells. The authors found that silencing COX-2 reduced EGFR expression following DCA stimulation, whereas silencing EGFR had a less pronounced effect on COX-2 levels. The observed asymmetric effects of COX-2 and EGFR knockdown on DCA-induced signaling are consistent with a bidirectional crosstalk: EGFR activation promotes COX-2 transcription (via MAPK/ERK and nuclear EGFR mechanisms),^22^ whereas COX-2 activity (through production of PGE₂) can transactivate EGFR via EP receptor–mediated Src activation and a disintegrin and metalloproteinase (ADAM)-dependent shedding of EGFR ligands.^21^ This positive feedback loop has been reported in several cancer models; however, its magnitude and whether it affects EGFR phosphorylation or EGFR expression may vary depending on the cellular context.^23^ Accordingly, the data were consistent with a model in which COX-2 may function as an amplifier of EGFR signaling in BDC cells under DCA stimulation.

The IGF-1R signaling cascade, which involves receptor activation, IRS-1 phosphorylation, and subsequent PI3K/Akt pathway activation, triggers the activation of mTOR, a master regulator of cellular protein synthesis and growth.^24^ Multiple factors, including EGFR and IGF-1R, can enhance PI3K/Akt signaling.^25^ In BDC, PI3K/Akt induction through COX-2 and EGFR activation is identified as a fundamental mechanism for promoting proliferation, invasion, and angiogenesis.^26^ Previous research from the authors demonstrated that metformin exhibited antineoplastic properties in BDC by both activating the AMPK^Thr172^/TSC-2 pathway and counteracting the IGF-1R/IRS-1/Akt-mediated inhibition of TSC-2, ultimately leading to the suppression of mTOR signaling.^12^ The data suggested that ASA uses similar mechanisms by interfering with IGF-1R/IRS-1–mediated suppression of TSC-2, thereby inhibiting mTOR signaling. The importance of this pathway was confirmed by the IGF-1R-silencing experiments, which resulted in increased TSC-2 signaling and downregulation of mTOR even in the absence of ASA treatment, suggesting that IGF-1R represents a crucial target for modulating BDC cell proliferation. Additionally, the results demonstrated that AMPK^Thr172^ phosphorylation plays a key role in regulating TSC-2 and mTOR expression in BDC cells. This finding was consistent with observations in pancreatic cancer, where metformin exerts antitumor effects through AMPK^Thr172^ phosphorylation via the LKB1 signaling pathway.^27^ The dual mechanism of ASA (i.e., targeting both the IGF-1R/IRS-1/TSC-2/mTOR axis and the AMPK^Thr172^/TSC-2 pathway) was associated with inhibition of BDC cell proliferation and represents a novel aspect of the antineoplastic effects of ASA on BDC.

EMT is a reversible process in which epithelial cells acquire mesenchymal characteristics, beginning with suppression of E-cadherin and breakdown of adherens junction.^28^^,^^29^ The loss of epithelial markers, notably E-cadherin, together with increased expression of mesenchymal markers, such as N-cadherin, Slug, and S100A4, is strongly associated with aggressive BDC features, including high metastatic potential, perineural and vascular invasion, poor differentiation, and more advanced disease stage.^30^^,^^31^ Moreover, EGFR activation destabilizes the E-cadherin/β-catenin complex in various tumor types, thereby disrupting intercellular adhesion, promoting EMT progression, and enhancing cellular motility.^24^^,^^32^^,^^33^ On the basis of these mechanisms and the findings regarding the ability of ASA to inhibit EGFR signaling, the authors hypothesized that ASA may suppress EMT, thereby reducing BDC aggressiveness. The experiments supported this hypothesis, showing that ASA treatment effectively restored E-cadherin expression suppressed by EGF stimulation while simultaneously reducing EGF-induced N-cadherin upregulation. These results suggest that ASA inhibits EMT primarily by disrupting the EGF–EGFR signaling axis, which may underlie its ability to reduce BDC invasiveness and metastatic potential.

The current study has several limitations. Although cell viability above 90% in the MTT assay is generally considered noncytotoxic,^34^ the 2.5 mM ASA concentration used exceeds clinically achievable plasma levels and therefore represents a pharmacological limitation. In the experiments, 2.5 mM ASA maintained 98% viability at 48 hours and 90% at 72 hours, whereas 10 mM ASA reduced viability to 85% and 70% at the same time points, respectively. To distinguish regulated apoptotic signaling from nonspecific cytotoxicity, the authors additionally performed apoptosis assays and Western blot analyses of apoptosis-related proteins. Furthermore, because in vitro models lack the complex systemic metabolism and long-term exposure found in in vivo models, higher concentrations are often used to clearly elucidate specific molecular mechanisms within a compressed 24- to 72-hour timeframe. Another limitation is that all experiments were conducted in a single BDC cell line (SNU-245). Although this model provides a controlled experimental background, it does not fully reflect the genetic heterogeneity of BDC, including frequent alterations in EGFR, p53, and KRAS. Therefore, further validation in additional BDC cell lines with diverse molecular characteristics, as well as in vivo models, will be necessary to determine the broader applicability of these findings. Ongoing studies are using multiple BDC cell lines with distinct mutational profiles and xenograft models to validate the signaling interactions identified in this study and to assess their translational relevance in more physiologically representative settings.

**Figure 5:**
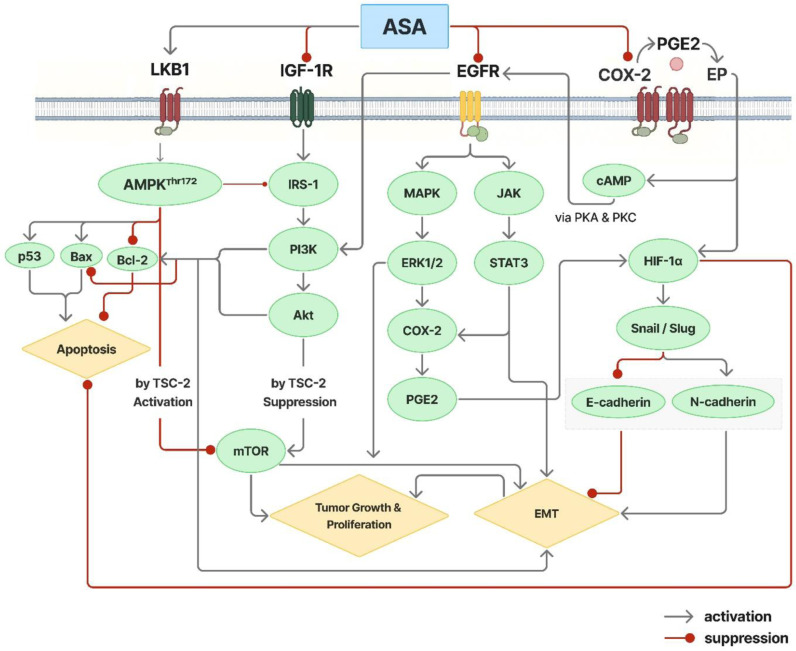
Proposed mechanisms of the antineoplastic effects of ASA on BDC cells, based on the findings of this study and a comprehensive review of the literature. (1) EGFR inhibition: ASA inhibits EGFR, which is involved in the activation of the MAPK pathway and signal transducer and activator of transcription 3 (STAT3)/Janus kinase (JAK) pathways that drive tumor growth, proliferation, and EMT.^35^ Additionally, EGFR-induced cytoplasmic COX-2 enhances prostaglandin E2 (PGE2) production, which in turn stimulates hypoxia-inducible factor-1α (HIF-1α), further promoting EMT and inhibiting apoptosis.^36^ (2) COX-2 inhibition: ASA suppresses membrane-bound COX-2 and E-prostanoid receptors (EP), sequentially downregulating HIF-1α expression and thereby contributing to EMT suppression.^36^ Furthermore, EP activation by COX-2 leads to increased cyclic adenosine monophosphate (cAMP) production via protein kinase A (PKA) and protein kinase C (PKC), which enhances EGFR expression.^37^ The current study is consistent with a reciprocal regulatory relationship, where EGFR appears to be required for full COX-2 activation and COX-2 appears to contribute to EGFR activation. (3) Inhibition of the IGF-1R: ASA suppresses IGF-1R/IRS-1/PI3K/Akt signaling cascade, a pathway that normally inhibits TSC-2 activity. This suppression leads to the downregulation of mTOR, thereby attenuating tumor growth. Moreover, PI3K and Akt inhibit apoptosis by upregulating Bcl-2 and downregulating Bax.^25^^,^^33^^,38^ (4) Activation of AMPK^Thr172^: ASA induces phosphorylation of AMPK at Thr172, leading to TSC-2-mediated inhibition of mTOR. This phosphorylation event also promotes apoptosis by increasing the expression of Bax and p53 while decreasing Bcl-2 levels in BDC cells.^27^ (5) Reversal of EMT: ASA reverses EMT by restoring E-cadherin expression and suppressing N-cadherin expression. These effects are mediated through the inhibition of both COX-2 and EGFR signaling.^31^^,^^39^ In addition, the suppression of PI3K, Akt, and mTOR by ASA also contributes to the inhibition of EMT.^40^ ASA, acetylsalicylic acid; BDC, bile duct carcinoma; COX-2, cyclooxygenase-2; EGFR, epidermal growth factor receptor; EMT, epithelial–mesenchymal transition; mTOR, mammalian target of rapamycin; PI3K, phosphoinositide 3-kinase.

In conclusion, this study showed that ASA treatment reduced BDC cell proliferation and induced coordinated changes in apoptosis-related proteins, COX-2/EGFR signaling, IGF-1R/IRS-1 activity, and AMPKThr172/TSC-2/mTOR regulation. ASA exposure was also associated with changes in E-cadherin and N-cadherin expression, suggesting a potential effect on EMT-related processes. The proposed signaling interactions are based on these in vitro observations and provide a mechanistic framework for further investigation (Figure 5). These findings provide a rationale for subsequent studies in more complex experimental systems to clarify the potential translational relevance of ASA in BDC.

## Supplementary Materials

Supplementary Material

## Data Availability

The data that support the findings of this study are available on request from the corresponding author.
