# Supplementary material for: Aspirin (Acetylsalicylic Acid) Exerts Antineoplastic Effects on Bile Duct Carcinoma Cells Through Modulation of COX-2/EGFR, AMPK, and IGF-1R Signaling Pathways
Source: Turk J Gastroenterol. 2026 May 4;37(6):722–31. doi: 10.5152/tjg.2026.25775 (PMC13247861; doi:10.5152/tjg.2026.25775)
Supplement: Supplementary Material [file supplementary_material.pdf]

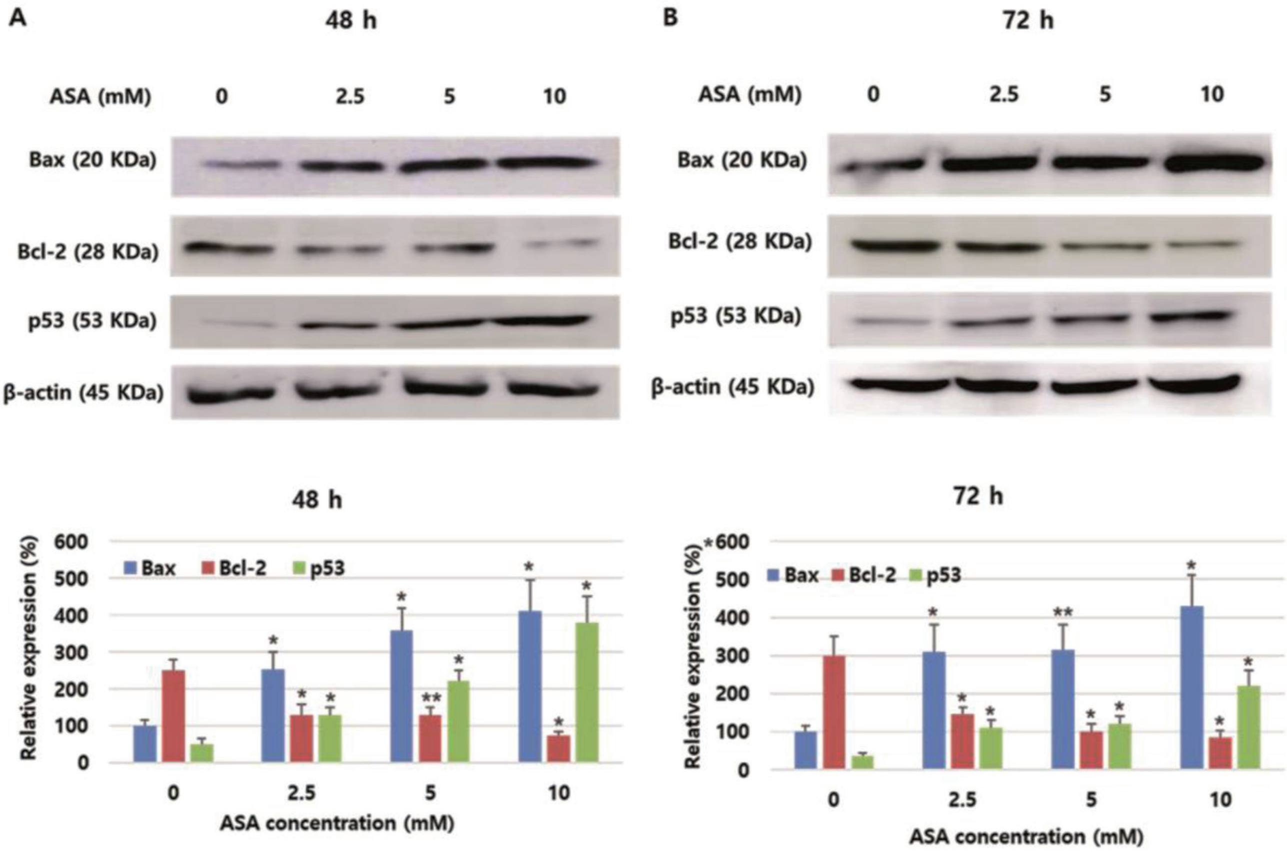

Supplementary Figure 1. ASA enhances the expression of Bax and p53 associated with apoptosis and tumor suppression and downregulates the expression of Bcl-2, which prevents apoptosis of BDC cells. Western blot analysis of Bax (pro-apoptotic), p53 (tumor suppressor), and Bcl-2 (anti-apoptotic) protein expression in BDC cells treated with ASA (0, 2.5, 5, and 10 mM) for 48 h (A) or 72 h (B). Representative blots are shown with quantification of protein levels normalized to β-actin. *P < .01 and **P < .01 vs. untreated control cells and cells treated with lower concentrations of ASA (for the respective proteins)

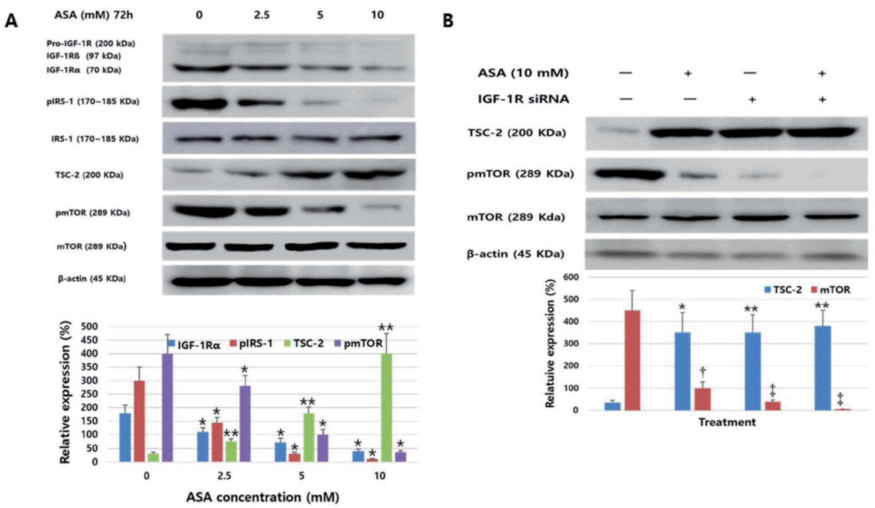

Supplementary Figure 2. ASA modulates the IGF-1R/IRS-1 pathway, which is associated with altered TSC-2 expression and reduced mTOR phosphorylation in BDC cells. (A) BDC cells were treated with ASA (0, 2.5, 5, and 10 mM) for 72 h. Protein expression levels of IGF-1R, IRS-1, TSC-2, and mTOR were analyzed using Western blot analysis. *P < .01 vs. untreated control cells and cells treated with lower concentrations of ASA (for IGF-1R, pIRS-1, and pmTOR); **P < .01 vs. untreated control cells and cells treated with lower concentrations of ASA (for TSC-2). (B) BDC cells were transfected with siRNA against IGF-1R for 4 h, followed by treatment with ASA (10 mM) for 72 h. TSC-2 and mTOR protein expression were analyzed using Western blot analysis. *P < .001 vs. untreated control for TSC-2; **P < .001 vs. untreated control (P > .05 vs. only ASA-treated cells without siRNA silencing) for TSC-2; $^{\dagger}P$ < .001 vs. untreated control for pmTOR; $^{\ddagger}P$ < .001 vs. untreated control and only ASA-treated cells for pmTOR expression.

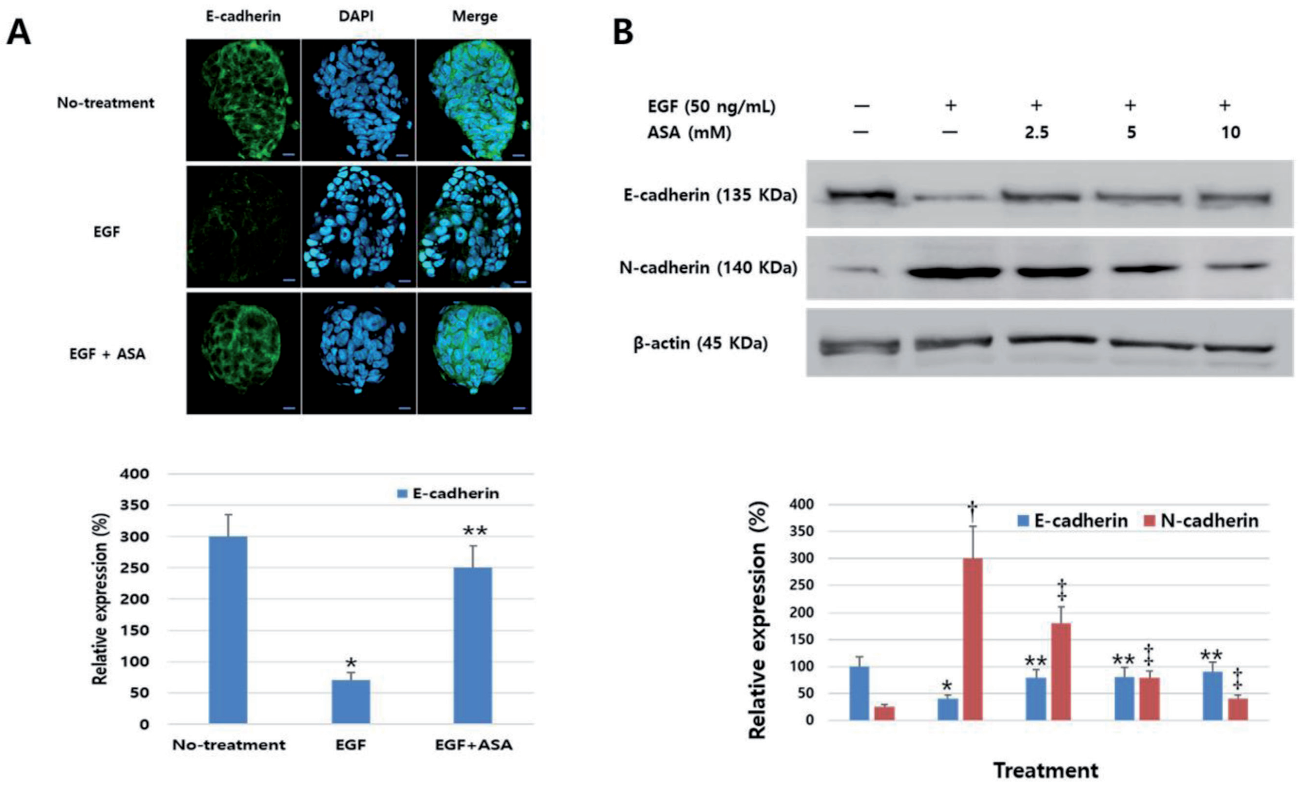

Supplementary Figure 3. ASA reversed EMT in BDC cells by restoring E-cadherin and N-cadherin expression. (A) Immunofluorescence staining of E-cadherin in BDC cells treated with ASA (10 mM) with or without EGF (50 ng/mL) in the medium for 72 h. ASA treatment restored E-cadherin expression suppressed by EGF stimulation. Nuclei were counterstained with DAPI (blue). Scale bar, 25 μm. Representative images from three independent experiments are shown. **P* < .001 vs. untreated control, ***P* < .001 vs. cells treated with EGF. (B) Western blot analysis of E-cadherin and N-cadherin expression in BDC cells treated with the indicated concentrations of ASA with or without EGF (50 ng/mL) for 72 h. **P* < .001 vs. untreated control for E-cadherin, ***P* < .001 vs. expression of E-cadherin treated with only EGF, $^{\dagger}P$ < .001 vs. untreated control for N-cadherin, $^{\ddagger}P$ < .01 vs. only EGF-treated and cells treated with lower concentrations of ASA for N-cadherin.
